# Clinical Efficacy and Safety of Acupressure on Low Back Pain: A Systematic Review and Meta-Analysis

**DOI:** 10.1155/2021/8862399

**Published:** 2021-02-24

**Authors:** Tao Li, Xiaohui Li, Fan Huang, Qiang Tian, Z. Y. Fan, S. Wu

**Affiliations:** ^1^The Second Clinical Medical College, Guangzhou University of Chinese Medicine, Guangzhou 510405, Guangdong, China; ^2^Massage Department, Guangdong Hospital of Traditional Chinese Medicine, Guangzhou 510405, Guangdong, China

## Abstract

**Objectives:**

To evaluate the effectiveness and safety of acupressure on low back pain (LBP).

**Methods:**

We searched 7 electronic databases and 2 trial registries through December 28, 2020. Randomized controlled trials (RCTs) of acupressure on LBP were considered for meta-analysis with Revman 5.3 and Stata 15.0 software. Methodological quality was evaluated using the Cochrane Collaboration's tool. Trial sequential analysis (TSA) was used to quantify the statistical reliability. HETRED analysis and GRADE were used to determine the heterogeneity and quality of the results, respectively.

**Results:**

Twenty-three RCTs representing 2400 participants were included. Acupressure was superior to tuina massage on response rate (RR 1.25; 95% CI, 1.16 to 1.35; *P* < 0.00001) and in the standardized mean difference (SMD) for pain reduction [SMD −1.92; 95% CI, −3.09 to −0.76; *P*=0.001]. Likewise, acupressure was superior to physical therapy [SMD, −0.88; 95% CI, −1.10 to −0.65; *P* < 0.00001] and to usual care [SMD, −0.32; 95% CI, −0.61 to −0.02; *P*=0.04] in pain reduction. The Oswestry Disability Index was significantly improved by acupressure compared with usual care [SMD, −0.55; 95% CI, −0.84 to −0.25; *P*=0.0003]. The combination of acupressure with either manual acupuncture or electro-acupuncture showed significant improvements over the adjuvant therapies alone in response rate [RR 1.19; 95% CI, 1.13 to 1.26; *P* < 0.00001], pain reduction, and the Japanese Orthopedic Association score (JOA). However, each study displayed substantial heterogeneity. Through subgroup sensitivity analysis and -HETRED analysis, the heterogeneity of acupressure compared with manual acupuncture decreased while the results maintained significance with respect to pain reduction [SMD −0.9; 95% CI, −1.21 to −0.6; *P* < 0.00001] and JOA [SMD, 0.66; 95% CI, 0.33 to 0.98; *P* < 0.00001]. Similar results were obtained comparing acupressure with electro-acupuncture with respect to pain [SMD, −1.07; 95% CI, −1.33 to −0.81; *P* < 0.00001] and JOA [SMD, 0.89; 95% CI, 0.51 to 1.27, *P* < 0.00001]. TSA demonstrated the effectiveness of acupressure as a standalone or as a combinative treatment (with manual acupuncture or electro-acupuncture) for LBP.

**Conclusion:**

Acupressure is an effective treatment for LBP. However, GRADE assessments downgraded the evidence in the trials, indicating that additional investigations are needed to confirm these observations.

## 1. Introduction

Low back pain (LBP) affects 540 million people worldwide and is the leading cause of disability [1]. The number of years lived with LBP has increased by 54% between 1990 and 2015 at all income levels and age groups worldwide [1]. In the USA, annual healthcare costs of LBP are estimated to be $100 billion, imposing an economic burden on the healthcare system [2]. An underlying cause for LBP has not been identified for most patients. However, several pathological causes have been identified for some patients such as intervertebral disc damage, vertebral fracture, tumors, infection, and physical and mental comorbidities [3]. Many therapeutic options are currently recommended for LBP, but the emphasis is now on complementary medicine such as exercise and physical therapy rather than on pharmacological and surgical treatments [4].

In traditional Chinese medicine (TCM), LBP is closely associated with the obstruction of the meridians and collaterals of the lower back, which start at the head, descending parallel to the midline and the lateral of the back, and end in the feet. The treatment is based on dredging the obtrusion of these meridians [5]. Acupressure on the meridians and collaterals is a noninvasive and straightforward technique, embracing the principle of Yin and Yang and the close relationship between Qi and blood circulation [6]. Acupressure from fingers, palms, or elbows on the appropriate acupoints helps promote the circulation of Qi and blood in the meridians of the lower back, relaxing muscles, and alleviating LBP [7]. Previous studies have suggested that acupressure may increase the production of endogenous sedatives and analgesics by the stimulation of the autonomic nervous system [8]. The efficacy of acupressure on LBP has been assessed in several meta-analyses. However, these reviews discussed the effectiveness of *auricular* acupressure on LBP based on limited researches or the acupressure treatments from nontherapists or the use of instrumentation [9–11]. We performed this analysis because of the absence of a meta-analysis on the therapeutic effects and safety of traditional acupressure on the effectiveness of traditional acupressure treatment of LBP.

## 2. Methods

### 2.1. Registration

The review was registered with the International Prospective Register of Systematic Reviews (PROSPERO) database (registration number: CRD42020144586).

### 2.2. Data Source and Search Strategy

The meta-analysis and systematic review were conducted following the Preferred Reporting Items for Systematic Reviews and Meta-Analysis (PRISMA) statement [12]. Literature searches were conducted through the following electronic databases from their inception to November 25, 2019: PubMed; Embase; the Cochrane Central Register of Controlled Trials (CENTRAL); Clinicaltrials.gov; the Chinese Scientific Journal Database (VIP); Wan-fang Data; the Chinese Biomedical Literature database (CBM); the China National Knowledge Infrastructure (CNKI); and the Chinese Clinical Trial Registry (ChiCTR). We searched MeSH term trees for “acupressure” and “low back pain” in PubMed, and the keywords searched included “acupressure”, “shiatsu”, “zhi ya”, “low back pain”, “lumbago”, “low back ache”, and “randomized controlled trial”. The above keywords were translated into Chinese and searched in the abovementioned Chinese databases as well. Search terms were combined with the Boolean “AND” and “OR” terms in search strategies, e.g., “acupressure” OR “shiatsu” OR “zhi ya”) AND (“low back pain” OR “lumbago” OR “low back ache”) AND (“randomized controlled trial” (Table 1 in the Supplement). Selected studies and reviews were screened for relevant trials. Journal editors and experts in the relevant fields were consulted for additional study sources. Finally, Open Grey was searched for qualifying studies.

### 2.3. Inclusion Criteria

#### 2.3.1. Types of Study

Randomized control trials (RCTs) that evaluated the effectiveness of acupressure on LBP were included. No restrictions were set on publication type, language, or status.

#### 2.3.2. Types of Participants

Patients (≥18 years old) who were diagnosed with LBP were eligible for inclusion without any restriction on sex, nationality, race, the period or nature of prior treatments, past or existing diseases, economic status, or inpatient or outpatient care.

#### 2.3.3. Types of Interventions

For interventions, acupressure only or adjunctive treatments, such as usual care, sham acupressure, acupuncture, or physical therapy, combined with acupressure was applied in the test group.

For control groups, usual care, sham acupressure, acupuncture, or physical therapy could be applied in the control group. If acupressure was applied with adjunctive treatments, the identical adjunctive treatment should be applied in the control groups as well.

#### 2.3.4. Types of Outcome Evaluations

Response rate, pain intensity, and functional ability were used as primary outcomes to evaluate the effectiveness of acupressure compared to other treatments. Response rate is defined as the proportion of participants who reported relief from pain or symptoms. Pain intensity was measured by the Visual Analogue Scale (VAS). Since VAS has either 0–10 cm or 0–100 mm range, the values are normalized up to a 0–10 cm range for comparability. For functional ability, we used the Oswestry disability index (ODI) and Japanese Orthopedic Association scores (JOA). The JOA has either 0–17 or 0–29 scores, and all 0–17 scales are normalized to 0–29 scores for comparability.

Adverse events during treatments and the usage count of each acupoint selected in the articles were analyzed as secondary outcomes.

### 2.4. Exclusion Criteria

Studies applying auricular acupressure or acupressure on specific reflexology areas of the hands or feet were excluded to study the effectiveness of acupressure on the meridians and collaterals of the body. Investigations in which acupressure was undertaken by therapeutic devices or non-medical staff were excluded as well.

### 2.5. Data Extraction

Data extraction was completed in duplicate and independently by two reviewers (TL and XL). Specific characteristics were extracted: author; publication year; sample size; the detail of the intervention and comparison; frequency; duration of treatments; follow-up intervals; and outcomes. We contacted the authors of the studies twice over six weeks via e-mail for missing or unclear data. If no response was received, the data were marked as unclear. A third reviewer (QT) assisted for consensus when disagreement occurred.

### 2.6. Assessment of Quality and the Level of Evidence

Methodological quality was assessed with the Cochrane Collaboration's tool for assessing risks of bias from 7 domains: random sequence generation; blinding of patients; allocation concealment; selective reporting; incomplete outcome data; and other biases [13]. Each of these domains was rated as low, high, or unclear risk. Each study was classified at one of three levels: low, high, or unclear risk of bias based on the overall risk assessments of the 7 domains. The quality of the body of evidence for the different outcomes was assessed with the GRADE approach (GRADE pro, Version 3.6 for Windows, Grade Working group) [14].

### 2.7. Statistical Analysis

We analyzed all outcomes with Stata 15.0 (Stata Corp, College Station, TX, USA). Studies were arranged according to the outcome measures and the type of intervention (acupressure monotherapy or in combination with acupuncture). Pooled dichotomous outcomes were presented as risk ratios (RRs) and 95% confidence intervals (CIs) while pooled continuous outcomes were presented as weight mean differences (WMDs) with 95% CIs or standardized mean differences (SMDs) with 95% CIs when using multiple measurements. The fixed-effect model was applied if heterogeneity was not significant (*I*^2^ ≤ 50% or *P* ≥ 0.10 in the *Q* test). Otherwise, subgroup analysis was performed to identify the source(s) of the heterogeneity. Planned subgroup analyses included the types of adjunctive interventions (manual acupuncture or electro-acupuncture) and the duration of treatments (≤2 weeks or > 2 weeks). Sensitivity analysis and HETRED analysis were conducted to further identify the trials resulting in significant heterogeneity [15]. If the source(s) of the heterogeneity was still unidentifiable, a random effect model was utilized for data synthesis.

#### 2.7.1. Publication Bias

Publication bias was assessed using funnel plots and Egger's test quantitatively when at least 10 trials were included. Significant publication bias was considered when *P* < 0.05.

#### 2.7.2. Trial Sequential Analysis

Trial sequential analysis (TSA) was conducted to detect the robustness of the results of the meta-analysis by calculating the required information size (RIS) with TSA 0.9.5.5 software (Copenhagen Trial Unit, Centre for Clinical Intervention Research, Rigshospitalet, Copenhagen, Denmark) [16]. We took the sample size as RIS. The required indexes for TSA were type I error (*α* = 0.05) and type II error (*β* = 0.2).

## 3. Results

### 3.1. Literature Search Results

**Figure 1:**
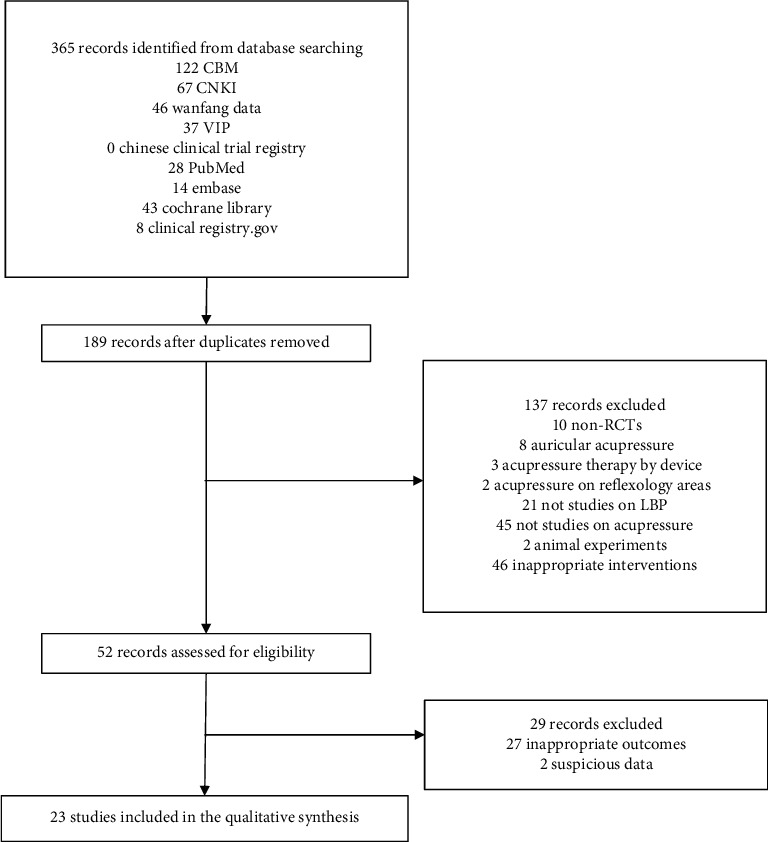
PRISMA flow diagram.

The Preferred Reporting Items for Systematic Reviews and Meta-analyses (PRISMA) flow diagram (Figure 1) shows the entire review process from the original search to the final selection of studies. Twenty-three studies [8, 17–38] with a total of 2400 participants were included. The characteristics of the included RCTs are presented in Table 1.

### 3.2. Characteristics of Interventions and Controls

Table 1 summarizes the characteristics *o*f 23 RCTs in this analysis. In seven studies [20, 23, 28, 33, 34, 36, 38], acupressure was compared with tuina massage (TM). In these studies, acupressure was distinguished from TM by the methods recorded, and no acupressure techniques were involved in TM in the control group. In three studies [18, 19, 26], acupressure was compared with physical therapy (PT). In two of these trials [18, 19], sample size, patient characteristics, and evaluation of outcomes differed but otherwise used similar research designs and interventions. This indicated that these studies were not duplicates. In seven studies, acupressure combined with electro-acupuncture was compared to electro-acupuncture alone [21, 25, 29–32, 37]. In four studies, acupressure combined with manual acupuncture was compared to manual acupuncture alone [22, 24, 27, 35]. In two of these studies [24, 31], the frequency and duration of the acupressure treatments differed between trials. In two studies [8, 17], acupressure was compared with usual care (UC), which included conventional medication or health education. In 17 studies [8, 20–23, 25–27, 29, 30, 32–38], acupressure was performed at least once a day. In three studies [31, 35, 37], acupressure combined with acupuncture was performed once every other day. In two studies [17, 28], acupressure was performed once a week. The duration of treatment periods among trials ranged from 2 weeks to 5 weeks (Table 1).

#### 3.2.1. LBP Outcomes

Response rates were measured in 17 studies [20–30, 32–38]. Values for JOA, VAS, and ODI were determined in 7 studies [21, 22, 24, 25, 30, 35, 39], 14 studies [18, 19, 21, 22, 24–28, 30, 33, 34, 38], and 2 studies [8, 17], respectively. Since other outcomes, such as Roland and Morris disability questionnaire, fatigue severity scale, McGill pain questionnaire, straight leg raising test, or functional independence measurement, were separately applied in one of the included studies, they cannot be pooled and analyzed.

### 3.3. Quality Assessment

**Figure 2:**
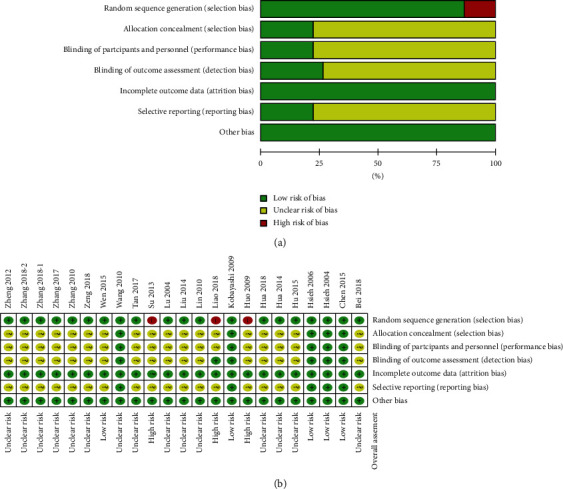
Risk of bias and summary.

Most of the 23 included studies reported proper random sequence generations (Figure 2(a)). There were 3 studies [23, 32, 37] that did not meet this requirement and were rated as having a high risk of bias for allocating patients according to their date of admission or clinic record number. There were 17 studies [20–27, 29–35, 37, 38] investigating the effectiveness of acupressure that (1) did not report an appropriate method for allocation concealment, (2) did not provide clear instructions for blinding of participants or personnel, or (3) lacked study protocols or available trial registration records (Figure 2(a)). These studies were also rated as having an unclear risk bias of selective reporting because of a lack of study protocols or available trial registration records. Only three studies [17–19] performed intention-to-treat analyses. All 23 studies were rated as having a low risk of attrition bias for incomplete outcome data (Figure 2(a)). Based on these results, the overall assessment of risks of bias was rated (Figure 2(b)). Three studies were classified at high risk of bias [23, 32, 37] while 5 studies [8, 17–19, 28] was with low risk of bias. The other 15 studies [20–22, 24–27, 29–31, 33–36, 38] were rated with unclear risk of bias.

### 3.4. Meta-Analysis

#### 3.4.1. Response Rate

**Figure 3:**
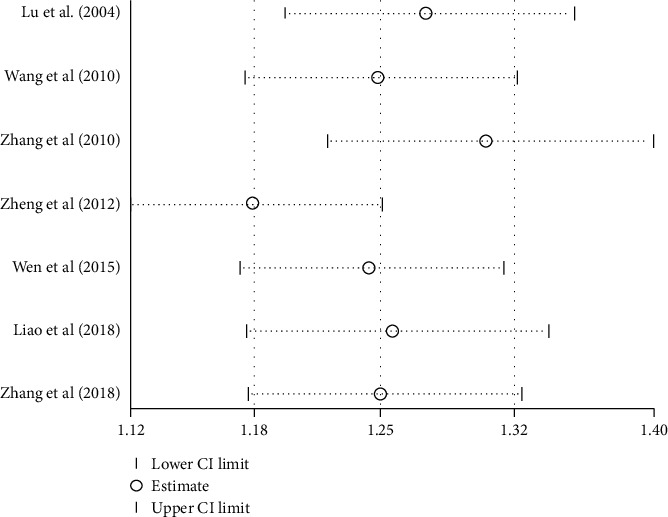
Sensitivity analysis of response rate of acupressure versus tuina massage.

**Figure 4:**
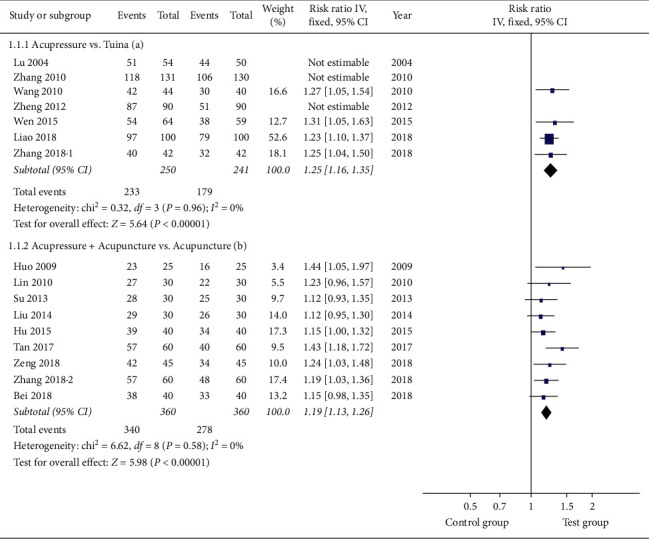
Forest plot of the response rates of acupressure versus tuina and acupressure + acupuncture versus acupuncture.

Seven studies [20, 23, 28, 33, 34, 36, 38] examined the response rate of acupressure on LBP compared with tuina massage. The meta-analysis assessed by a fixed-effect model revealed a significant difference in the short-term response rate, however with marked heterogeneity (*I*^2^ = 72%). Sensitivity analysis and HETRED analysis were undertaken (Figure 3). After removing the data of Lu et al. [38], Zhang [34], and Zheng et al. [33], the *I*^2^ value decreased from 72% to 0%. The pooled result in the fixed model (Figure 4(a)) was still in favor of acupressure [RR = 1.25; 95% CI, 1.16 to 1.35, *P* < 0.0001; heterogeneity, *I*^2^ = 0%, *P*=0.96].

Nine studies [21, 22, 25, 27, 29, 30, 32, 35, 37] reported a response rate the combinative therapy of acupressure with acupuncture in comparison with acupuncture alone. We found a significant difference in the short-term response rate with low heterogeneity [RR = 1.19; 95% CI, 1.13 to 1.26; *P* < 0.0001; heterogeneity, *I*^2^ = 0%; *P*=0.58] (Figure 4(b)).

#### 3.4.2. Pain Intensity

**Figure 5:**
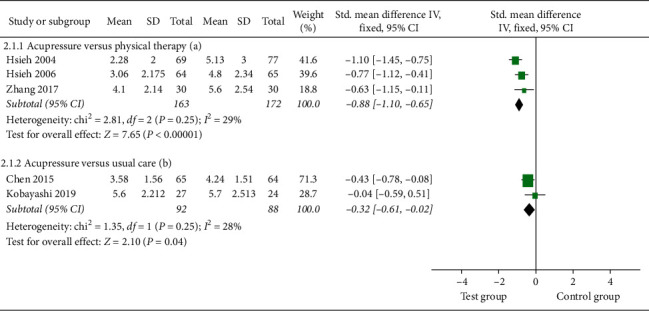
Forest plot of the pain intensity of acupressure versus physical therapy and acupressure versus usual care.

Three studies [18, 19, 26] compared the pain reduction of acupressure versus physical therapy at one month. There was evidence of significant pain relief using acupressure [SMD = −0.88; 95% CI, −1.110 to −0.65; *P* < 0.0001; heterogeneity, *I*^2^ = 29%; *P*=0.25] (Figure 5(a)).

Pooled results from two studies [8, 17] indicated that acupressure generated significant improvement on LBP compared to UC [SMD = −0.32; 95% CI, −0.61 to −0.02; *P*=0.04; heterogeneity, *I*^2^ = 26%; *P*=0.25] (Figure 5(b)).

**Figure 6:**
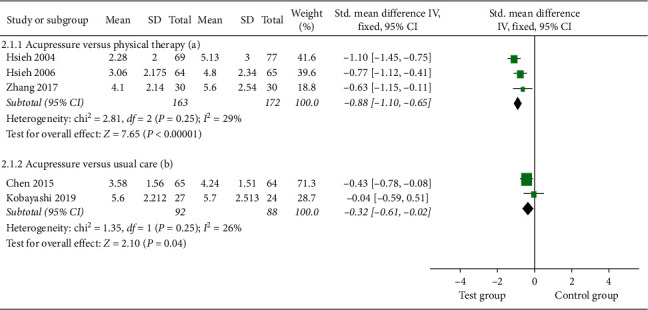
Forest plot of pain intensity of acupressure versus tuina massage.

Four studies [28, 33, 34, 38] assessed the analgesic effect of acupressure compared with tuina massage. The pooled data suggested a greater pain reduction, although with substantial heterogeneity (*I*^2^ = 97%). Owing to the similar clinical characteristics in these studies, we were unable to perform subgroup analysis. Sensitivity and HETRED analysis detected no potential source of heterogeneity. After confirming the data accuracy with the studies' authors, we adopted the random effect model and the subsequent analysis demonstrated favorable effects of acupressure [SMD, −1.92; 95% CI, −3.09 to −0.76; *P*=0.001] (Figure 6).

**Figure 7:**
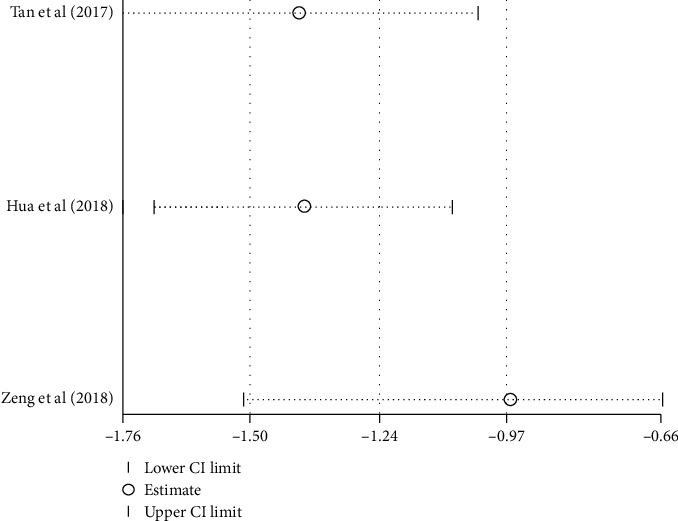
Sensitivity analysis of pain intensity of acupressure combined with manual acupuncture versus manual acupuncture.

**Figure 8:**
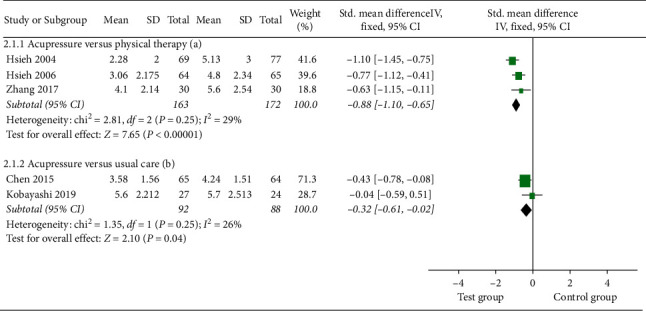
Forest plot of the pain intensity of acupressure combined with electro-acupuncture versus electro-acupuncture and acupressure combined with manual acupuncture versus manual acupuncture.

Six studies [21, 22, 24, 25, 30, 35] reported the effect of combination therapy of acupressure and acupuncture compared with acupuncture alone on pain intensity. Less pain intensity was indicated when using the combined treatment of acupressure and acupuncture [SMD, −1.13; 95% CI, −1.31 to −0.94; *P* < 0.00001]. However, since significant heterogeneity existed among these trials (*I*^2^ = 68%), a subgroup analysis was performed (Table 2). In the subgroup, the pooled result favored the combinative therapy of acupressure and electro-acupuncture with low heterogeneity [SMD, −1.07; 95% CI, −1.33 to −0.81; *P* < 0.00001; heterogeneity, *I*^2^ = 0%; *P*=0.37] (Figure 8(a)). In the subgroup for acupressure with manual acupuncture, the heterogeneity remained considerable (85%). Sensitivity and HETRED analyses were therefore performed (Figure 7). After removing the study from Zeng and Zhao [21], the *I*^2^ value decreased (85% to 2%) (Figure 8(b)). The subgroup analysis showed the combination of acupressure with manual acupuncture generated superior analgesic effects to the control group [SMD, −0.9; 95% CI, −1.21 to −0.6; *P* < 0.00001]. (Figure 8(b)).

#### 3.4.3. Japanese Orthopedic Association Score (JOA)

**Figure 9:**
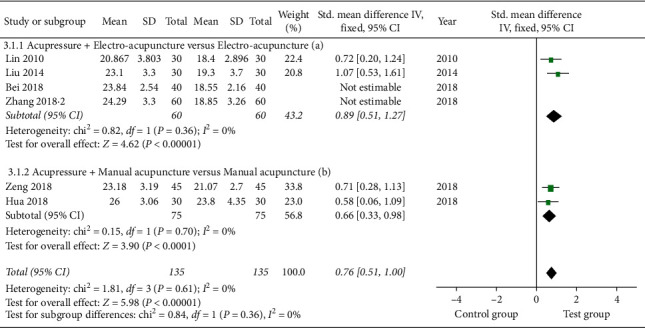
Forest plot of JOA.

**Figure 10:**
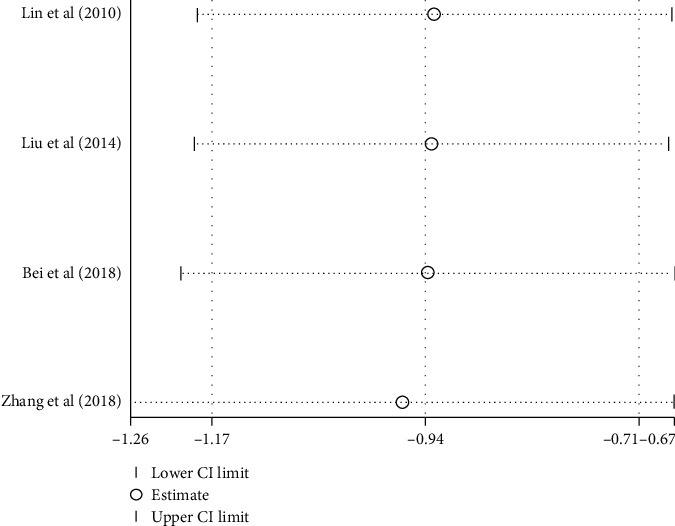
Sensitivity analysis of JOA: acupressure combined with electro-acupuncture versus electro-acupuncture.

The effects of acupressure plus acupuncture compared to acupuncture on LBP using JOA in 6 studies [21, 22, 24, 25, 30, 35] were examined. Because substantial heterogeneity was found in these studies (*I*^2^ = 84%), we performed a subgroup analysis based on the duration of treatments or types of adjunctive interventions (Table 2). In the subgroup for the combination of acupressure with manual acupuncture, the pooled data in fixed model demonstrated a significant increase of JOA scores [SMD, 0.66; 95% CI, 0.33 to 0.98); *P* < 0.0001; heterogeneity, *I*^2^ = 0%; *P*=0.70] (Table 2 and Figure 9(b)). In the subgroup of acupressure with electro-acupuncture compared with electro-acupuncture alone, the heterogeneity was still considerable (*I*^2^ = 83%). The following sensitivity and HETRED analysis indicated that the inclusion of two studies [21, 25] might be the cause of the high heterogeneity (Figure 10). After removing these studies from the analysis, the pooled results in the fixed model showed a significant improvement in the test group [SMD, 0.89, 95% CI, 0.51 to 1.27; *P* < 0.00001; heterogeneity, *I*^2^ = 0%; *P*=0.36] (Figure 9(a)).

#### 3.4.4. Oswestry Disability Index (ODI)

**Figure 11:**
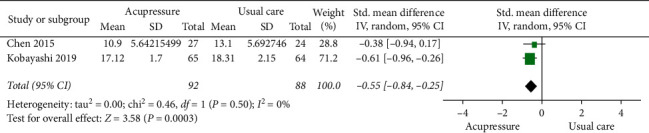
Forest plot of ODI

Two studies [8, 17] examined the effects of acupressure versus usual care on LBP using ODI. The pooled data suggested a significant improvement associated with acupressure compared with usual care [SMD, −0.55; 95% CI, −0.84 to −0.25; *P*=0.0003; heterogeneity, *I*^2^ = 0%; *P*=0.50] (Figure 11).

#### 3.4.5. Adverse Events

Adverse events reported in studies were sparse. Six studies [17, 19, 22, 32, 33, 35] mentioned the term “adverse reactions” of which 4 studies [19, 22, 33, 35] only descriptively reported that no adverse reaction occurred in either the test or control groups. Two studies [17, 32] reported the total number of the symptoms and the solutions to adverse events. The incidence of adverse reactions was 0.42% (5/1200) in the test groups compared with 0.25% (3/1200) in the control groups. The primary adverse reactions observed in the treatment group included muscle pain or headache while those in the control group included dizziness, urticaria, and abdominal pain. The adverse events of the two groups were tolerable and did not require specific interventions.

#### 3.4.6. Usage Counts of the Acupoints

**Figure 12:**
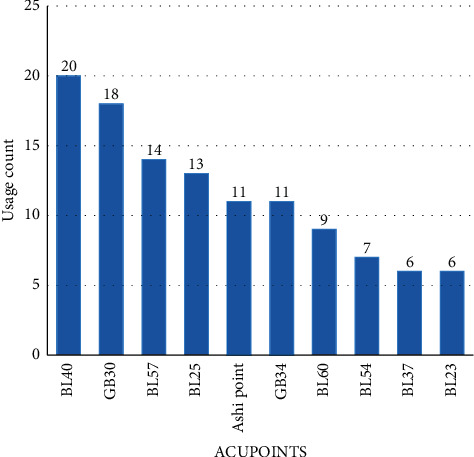
Comparison of acupoint use.

The usage counts of each acupoint selected for treatment were calculated. The most frequently-used acupoints were *Weizhong* (BL40), *Huantiao* (GB30), *Chengshan* (BL57), *Dachangshu* (BL25), *Ashi* points, *Yanglingquan* (GB34), *Kunlun* (BL60), *Zhibiao* (BL54), *Yinmen* (BL37), and *Shenshu* (BL23) (Figure 12).

#### 3.4.7. Trial Sequential Analysis (TSA)

**Figure 13:**
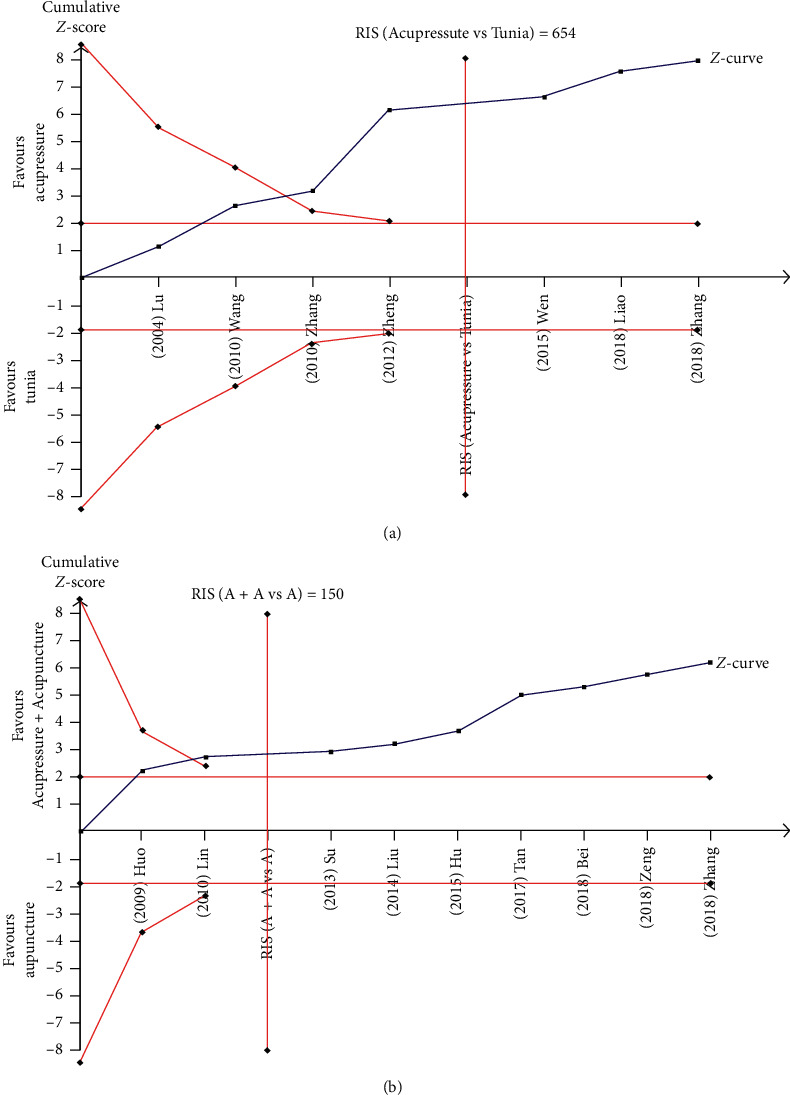
Trial sequential analysis on response rate comparing acupressure versus tuina massage and acupressure + acupuncture versus acupuncture.

TSA was undertaken with the data from 2 meta-analyses. A TSA on the comparison of the response rate of acupressure with that of tuina massage revealed that the cumulative *Z*-curve crossed the traditional boundary of 5% significance (horizontal line) and the monitoring boundaries (inward sloping curves) (Figure 13(a)). After the inclusion of Wang 2010, the significance of 5% was reached every time a new trial was added to the meta-analysis. The results support the conclusion that acupressure improves the response rate over that of tuina massage with RR = 1.25.

In the comparison between acupressure plus acupuncture versus acupuncture, TSA revealed that the cumulative *Z*-curve crossed the traditional boundary of 5% significance and the monitoring boundaries (Figure 13(b)), indicating that the sample size achieved the required 150 participants and confirmed that the combinative treatment could improve the response rate on LBP (RR = 1.19).

#### 3.4.8. GRADE

The grade of the evidence obtained for the response rate using JOA, VAS, or ODI for acupressure was low or very-low (Table 3). These results were similar to the grade of evidence for the combinative treatment of acupressure and acupuncture. Only the trials comparing acupressure with physical therapy provided moderate-grade evidence (Table 3).

The reasons for downgrading the evidence were the poor methodological quality, high heterogeneity, wide confidence intervals, and insufficient sample size among relevant trials. Wide confidence intervals and small sample sizes also contributed to the downgrading of the evidence. No serious indirectness was identified. The summary of findings and evidence profile is presented in Table 3.

## 4. Discussions

The systematic review and meta-analysis included 23 RCTs with 2400 participants with LBP. Consistent with previous systematic reviews [6, 40], moderate-quality evidence revealed an association between acupressure and greater pain relief compared with physical therapy. Although rated as very-low to low, poor quality evidence suggested that acupressure, with or without combinative acupuncture therapy, contributed to a greater amelioration of pain and functional disability from LBP compared with usual care, tuina massage, or acupuncture. TSA results revealed that adequate studies supported the significance of the clinical response rate of acupressure, with or without combinative acupuncture therapy, compared to other treatments.

Although positive results suggesting acupressure as a standalone or as a combinative treatment for LBP, high-quality evidence was insufficient to make an informed decision. The methodological limitations of the included RCTs may have impacted the accuracy and reliability of the evidence. Owing to the complex and variable characteristics of therapies like acupressure, acupuncture, and massage, most studies used a nonblinded pragmatic trial to study the efficacy of the therapies, which may have increased their performance bias. Rigorous research methods, such as sham-controlled double-blinded designs, are necessary to minimize bias in evaluating the effectiveness of acupressure. Most outcome measures were dependent on patient-reported scales or questionnaires (JOA, ODI, and VAS), which are vulnerable to the subjective conditions of the patient and/or assessor. Physiological outcome indicators, including the range of motion, pain threshold, and muscle tone in the lower back, are encouraged to be included in future planned research protocols. Only 3 RCTs conducted mid-to-long-term follow-up indicating that the long-term efficacy of acupressure remains to be considered.

The most frequently-used acupoints were located on the bladder and the gallbladder meridians. In TCM theory, these acupoints are closely correlated with the liver and kidney, which govern the function of bone and tendon. Acupressure over these points could promote the *Qi* and blood circulation of these specific meridians to improve the body's ability to recover the proper functions of muscles, tendons, and bones in the lower back [19]. Modern medical studies have suggested stimulation of acupoints could enable the production of endogenous opioids (endorphin) and certain peptides, which act as both analgesics and sedatives and alleviate LBP [41–43].

When acupressure was compared solely or as an adjunctive with acupuncture, substantial heterogeneity was found among the study's results. We conducted subgroup analyses on the duration of treatment and adjunctive interventions and found that adjunctive interventions might be contributing to the heterogeneity. However, considerable heterogeneity remained unexplained in the pooled results. In the comparison between acupressure and Tuina massage on pain intensity, the heterogeneity was substantial and unexplained. Acupressure is sometimes considered a subapplication of tuina massage since both are noninvasive techniques. However, the locations of interventions and operational techniques between the two forms are different. Tuina massage includes different techniques such as rolling, pushing, shaking, kneading, scrubbing, or stretching. We speculate that details of the tuina techniques, such as types, strength, angle, and duration of each movement, as well as details of the treatments, such as frequency and duration of the treatment, might result in the heterogeneity found in our analysis. Recording these details could improve assessments of the effectiveness of acupressure, but few studies have collected such information.

Sparse and mild adverse events were reported in the RCTs, consistent with findings in the previous reviews [6, 40, 44]. However, the unclear methodological protocols for assessing adverse events, the unclear predictors of these events, and the limited number of RCTs reporting them contribute to a low overall apparent incidence of adverse events. Therefore, therapists need to fully inform patients of the potential risks of adverse events and to pay close attention to their potential occurrence.

The most important limitation in this systematic review was the limited number of studies with a low risk of bias. Only 5 eligible RCTs (22%) were rated as low risk of bias in the overall assessment. Most of the studies included in this review were evaluated as “high” or “unclear” risk of bias. We did not conduct a funnel plot and Egger's test to detect publication bias since there were fewer than 10 studies in each comparison.

Although analysis of combinative MA and EA individually suggested that each was superior to acupuncture alone, the combinative therapies that included acupressure combined with acupuncture in test groups had high heterogeneity, making it difficult to determine the efficacy of acupressure. The outcome measures could have had high heterogeneity because most of the RCTs used patient-reported scales or questionnaires, which could be influenced by memory or emotional bias, undermining the reliability of the results. Moreover, there is no study investigating the effectiveness of acupressure on acute low back pain. Most studies included patients with chronic low back pain, and no clear distinction of patients' histories was made in the remaining studies.

## 5. Conclusion

We demonstrated that acupressure could provide clinical benefits to LBP conditions and had a significant short-term response rate in LBP management. However, the overall reliability of this conclusion is limited by the methodological quality of the included trials. Better designed large-scale RCTs using reliable quantitative methods are necessary to confirm the efficacy of acupressure for LBP.

## Figures and Tables

**Table 1:** Table 1Characteristics of the included studies.

Study	Sample size (T/C)	Age (years; SD) (T/C)	Intervention (T/C)	Frequency	Treatment duration	Follow-up interval		Outcome	
Hsieh et al. 2004 [18]	69/77	47.6 ± 13.6/47.6 ± 14.9	A/PT	Unclear	1 month	6 months		②⑥⑧	
Hsieh et al. 2006 [19]	64/65	50.2 ± 13.8/52.6 ± 17.2	A/PT	Unclear	1 month	6 months		③⑥	
Zhang et al. 2017 [26]	30/30	18 to 55	A/PT	QD	1 month	NM		①⑤⑥	
Wen 2015 [28]	64/59	52.72 ± 9.27/49.09 ± 14.31	A/TM	QW	5 weeks	NM		①⑥	
Zheng et al. 2012 [33]	90/90	45.6/46.2	A/TM	QD 5 times/week	20 days	1 year		①⑤⑥	
Lu et al. 2004 [38]	54/50	47.0 ± 10.41/45.0 ± 11.01	A/TM	QD	20 days	NM		①⑥	
Wang 2010 [36]	44/40	43.5 ± 10.5/42.8 ± 9.7	A/TM	QD	10 days	NM		①	
Liao et al. 2018 [23]	100/100	38.26 ± 7.65/38.37 ± 7.35	A/TM	QD	2 weeks	NM		①⑩	
Zhang et al. 2018 [20]	42/42	43.63 ± 3.23/42.95 ± 3.54	A/TM	QD	1 month	NM		①⑪	
Zhang 2010 [34]	131/130	43.36 ± 11.43/41.84 ± 11.62	A/TM	QD	20 days	NM		①⑥
Huo 2009 [37]	25/25	48.5/47.7	A + EA/EA	QOD	24 days	NM		①⑥
Liu and Guo 2014 [30]	30/30	44.15/46.23	A + EA/EA	QD 5 times/week	2 weeks	NM		①⑤
Hu 2015 [29]	40/40	40.2 ± 3.8/39.6 ± 3.5	A + EA/EA	QD	2 weeks	NM		①
Bei 2018 [25]	40/40	27.73 ± 3.51/28.16 ± 3.49	A + EA/EA	QD 5 times/week	2 weeks	NM		①⑥
Zhang and Zhao 2018 [21]	60/60	48.55 ± 10.28/48.58 ± 10.26	A + EA/EA	QD 6 times/week	3 weeks	NM		①⑤⑥
Hua et al. 2014 [31]	30/30	39.53 ± 9.66/41.93 ± 9.71	A + EA/EA	QOD	10 days	NM		⑥
Su 2013 [32]	30/39	52.87 ± 8.86/48.86 ± 11.24	A + EA/EA	QD 6 times/week	2 weeks		NM	①④⑧
Tan 2017 [27]	60/60	49.3 ± 8.5/50.1 ± 7.9	A + MA/MA	QD	20 days		NM	①④⑥
Hua and Wang 2018 [24]	30/30	43.6 ± 10.4/40.9 ± 9.7	A + MA/MA	QOD	10 days		NM	⑤⑥
Zeng and Pei 2018 [22]	45/45	38.05 ± 6.21/37.56 ± 5.68	A + MA/MA	QD 6 times/week	2 weeks		NM	①⑤⑥
Lin 2010 [35]	30/30	43.9 ± 9.922/44.6 ± 8.315	A + MA/MA	QOD	10 days		NM	①⑤
Kobayashi et al. 2019 [17]	27/24	67.4 ± 12.2/68.3 ± 15	A/UC	QW	1 month		1 month	③④⑥⑧
Chen 2015 [8]	65/64	18.75 ± 1.74/18.73 ± 0.63	A/UC	BID	1 week		4 months	④⑥

Note: (1). A: acupressure; PT: physical therapy; TM: tuina massage; EA: electro-acupuncture; MA: manual acupuncture; UC: usual care. (2). Outcome indicator: ① response rate; ② Short-Form Pain Questionnaires; ③ Roland and Morris Disability Questionnaire; ④ Oswestry Disability Index; ⑤ Japanese Orthopedic Association Score; ⑥ Visual Analogue Scale; ⑦ Fatigue Severity Scale; ⑧ Short-Form McGill Pain Questionnaire; TCM Syndrome Index; ⑨ straight leg raise; ⑩ functional independence measurement; (3). BID: twice a day; QD: once a day; QOD: once every other day; QW: once a week; NM: not mentioned.

**Table 2:** Subgroup analyses of the combination therapy of acupressure with acupuncture.

Characteristic	Patients	Studies	Fixed-effects SMD (95% CI)	Heterogeneity	
				*I* ^2^	*P*
*Pain intensity*					
Duration of treatments					
≤ 2 weeks	410	5	−1.16 (−1.37, −0.95)	74%	0.004
> 2 weeks	120	1	−1.02 (−1.40, −0.64)	—	—
Adjunctive interventions					
Manual acupuncture	270	3	−1.19 (−1.45, −0.92)	85%	0.001
Electro-acupuncture	260	3	−1.07 (−1.33, −0.81)	0%	0.37

*JOA*					
Duration of treatments					
≤2 weeks	410	5	1.22 (1.0, 1.43)	94.9%	<0.00001
>2 weeks	60	1	0.72 (0.20, 1.24)	—	—
Adjunctive interventions					
Manual acupuncture	150	2	0.66 (0.33, 0.98)	0.0%	0.7
Electro-acupuncture	320	4	1.43 (1.18, 1.68)	83.0%	0.0006

**Table 3:** GRADE summary of finding.

Interventions	Outcomes	Illustrative comparative risks (95% CI)		Relative effect (95% CI)	Number of participants (studies)	Quality of the evidence (GRADE)
		Assumed risk (control group)	Corresponding risk (test group)			
Acupressure versus tuina massage	Response rate	**743 per 1000**	**928 per 1000** (862 to 1000)	**RR 1.25** (1.16 to 1.35)	491 (5 studies)	⊕⊕⊝ **low**^a,b^
	VAS	The mean visual analog scale in the control groups was **4.3275 points**	The mean VAS in the intervention groups was **1.94 standard deviations lower** (0.78 to 3.11 lower)		663 (4 studies)	⊕⊝⊝⊝ **very low**^a,b,c^

Acupressure versus physical therapy	VAS	The mean visual analog scale in the control groups was **5.176 points**	The mean VAS in the intervention groups was **0.88 standard deviations lower** (1.10 to 0.65 lower)		335 (3 studies)	⊕⊕⊕⊝ **moderate**^d^

Acupressure versus usual care	VAS	The mean visual analog scale in the control groups was **4.97 points**	The mean VAS in the intervention groups was **0.32 standard deviations lower** (0.02 to 0.61 lower)		180 (2 studies)	⊕⊕⊝⊝ **low**^c,d^
	ODI	The mean ODI in the control groups was **15.705 points**	The mean ODI in the intervention groups was 0.55 **standard deviations lower** (0.25 to 0.84 lower)		180 (2 studies)	⊕⊕⊝⊝ **low**^c,d^

Acupressure + acupuncture versus acupuncture	Response rate	**772 per 1000**	**919 per 1000** (873 to 973)	**RR 1.19** (1.13 to 1.26)	720 (9 studies)	⊕⊕⊝⊝ **low**^a,b^

Acupressure + acupuncture versus acupuncture	VAS	The mean VAS in the control groups was **1.39 points**	The mean VAS in the intervention groups was **1.13 standard deviations lower** (0.94 to 1.31 lower)		530 (6 studies)	⊕⊝⊝⊝ **very low**^a,b,c^
	JOA	The mean JOA in the control groups was **19.095 points**	The mean JOA in the intervention groups was 1.14 **standard deviations higher** (0.94 to 1.34 higher)		470 (6 studies)	⊕⊝⊝⊝ **very low**^a,b,c^

Note: (1) The basis for the **assumed risk** (e.g., the median control group risk across studies) is provided in footnotes. The **corresponding risk** (and its 95% confidence interval) is based on the assumed risk in the comparison group and the **relative effect** of the intervention (and its 95% CI). (2) GRADE Working Group grades of evidence: **high quality:** further research is very unlikely to change our confidence in the estimate of effect; **moderate quality:** further research is likely to have an important impact on our confidence in the estimate of effect and may change the estimate; **low quality:** further research is very likely to have an important impact on our confidence in the estimate of effect and is likely to change the estimate; **very low quality:** we are very uncertain about the estimate. (3) a: randomization and blinding are not adequate or appropriate; b: high heterogeneity; c: confidence intervals are too wide; d: insufficient sample size.

## Data Availability

The data supporting this systematic review are from previous studies and datasets, which have been cited. The processed data are available from the corresponding author upon request.

## Supplementary Materials

Supplementary Materials Supplementary file 1: searching strategy for each database used in this review. Supplementary file 2: PRISMA checklist.
