# Supplementary material for: Clinical Efficacy and Safety of Acupressure on Low Back Pain: A Systematic Review and Meta-Analysis
Source: Evid Based Complement Alternat Med. 2021 Feb 24;2021:8862399. doi: 10.1155/2021/8862399 (PMC7932783; doi:10.1155/2021/8862399)
Supplement: Supplementary Materials — Supplementary file 1: searching strategy for each database used in this review. Supplementary file 2: PRISMA checklist. [file 8862399.f1.zip › 8862399.f1/0213 Updated eTable1 Details of Searching Strategy.docx]

eTable 1 Details of literature Search Strategy

# 1. Searching strategy in Pubmed:

# 1.1 Searching for literatures related to acupressure

# 1.1.1 MeSH: Acupressure

# 1.1.2 Entry Terms: Shiatsu; Zhi Ya; Chih Ya; Shiatzu: 1140 Results

#
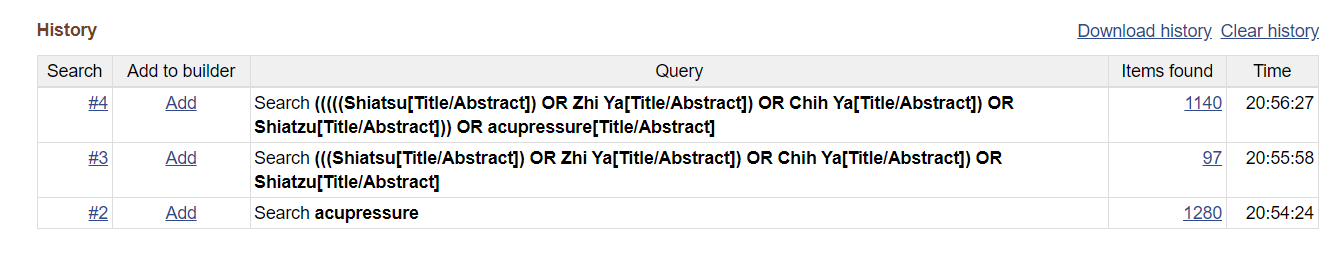


# 1.2 Searching for literatures related to low back pain: 38823 Results

#
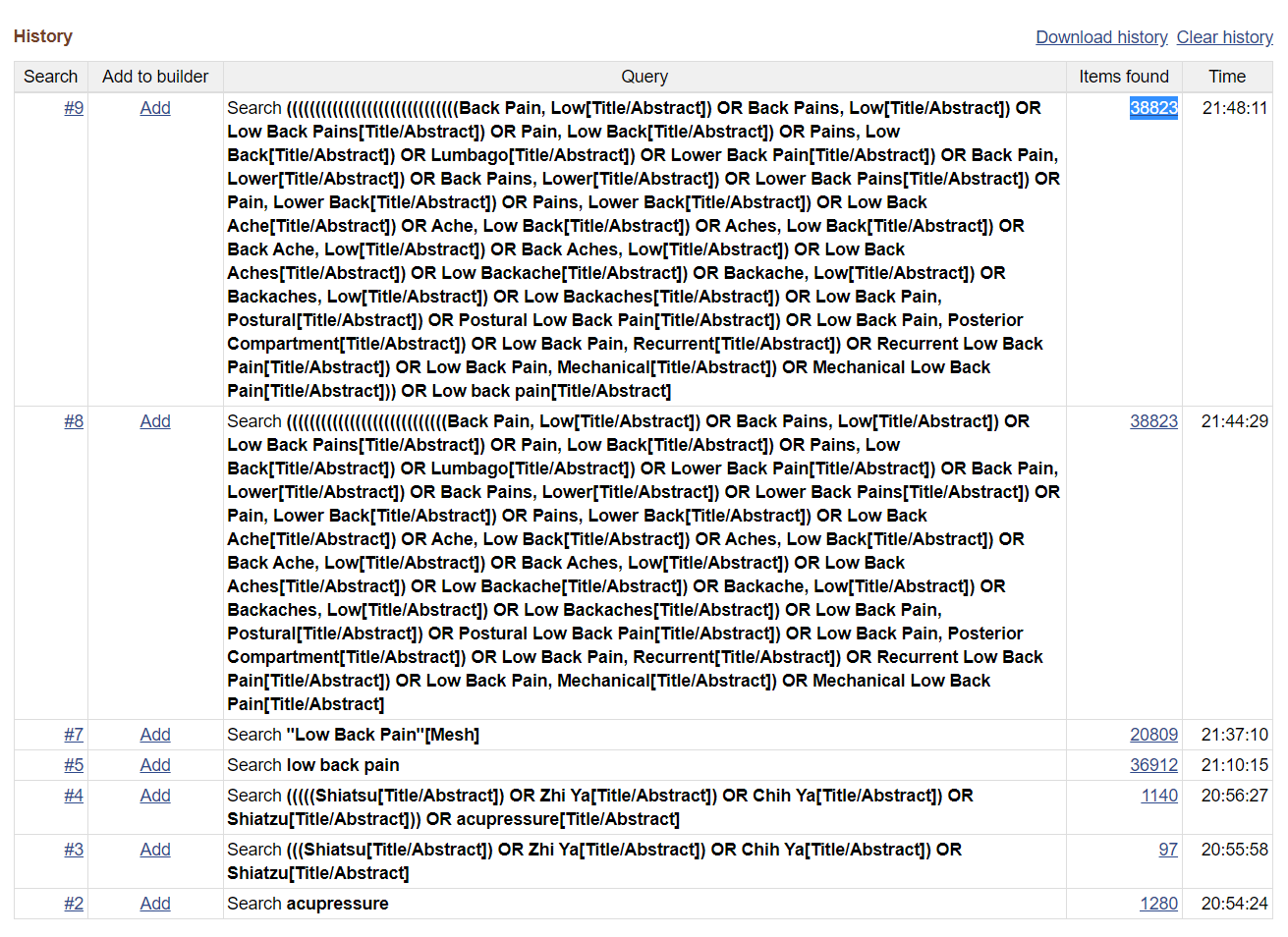


# 1.3 Searching for literatures related to acupressure or low back pain: 49 Results

#
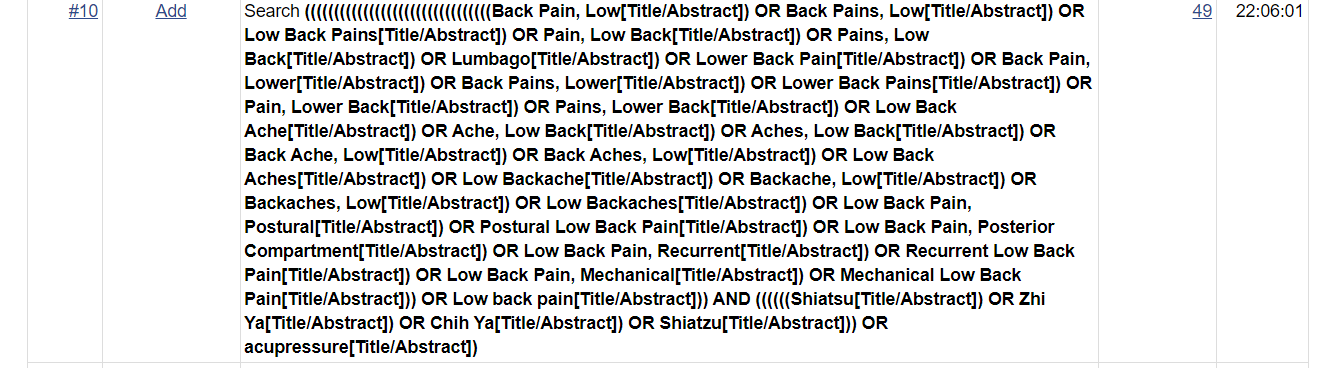


# 1.4 Searching for RCTs related to acupressure or low back pain: 27 results

#
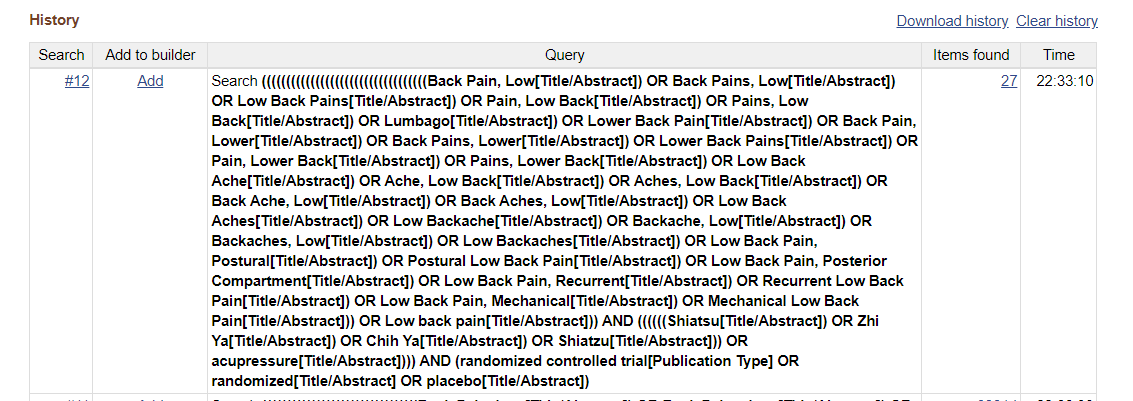


# 2. Searching strategy in Embase

# 2.1 Steps and details of literature search in Embase

#1 Search 'acupressure'/exp: 2,065 results

#2 Search 'Shiatsu':ab,ti OR 'Zhi Ya':ab,ti OR 'Chih Ya':ab,ti OR 'Shiatzu':ab,ti: 131 results

#3 Search 'acupressure'/exp OR 'Shiatsu':ab,ti OR 'Zhi Ya':ab,ti OR 'Chih Ya':ab,ti OR 'Shiatzu':ab,ti: 2,110 results

#4 Search 'low back pain'/exp: 55,017 results

#5 Search 'Back Pain, Low':ab,ti OR 'Back Pains, Low':ab,ti OR 'Low Back Pains':ab,ti OR 'Pain, Low Back':ab,ti OR 'Pains, Low Back':ab,ti OR 'Lumbago':ab,ti OR 'Lower Back Pain':ab,ti OR 'Back Pain, Lower':ab,ti OR 'Back Pains, Lower':ab,ti OR 'Lower Back Pains':ab,ti OR 'Pain, Lower Back':ab,ti OR 'Pains, Lower Back':ab,ti OR 'Low Back Ache':ab,ti OR 'Ache, Low Back':ab,ti OR 'Back Ache, Low':ab,ti OR 'Aches, Low Back':ab,ti OR 'Back Aches, Low':ab,ti OR 'Low Back Aches':ab,ti OR 'Low Backache':ab,ti OR 'Backache, Low':ab,ti OR 'Backaches, Low':ab,ti OR 'Low Backaches':ab,ti OR 'Low Back Pain, Postural':ab,ti OR 'Postural Low Back Pain':ab,ti OR 'Low Back Pain, PosteriOR Compartment':ab,ti OR 'Low Back Pain, Recurrent':ab,ti OR 'Recurrent Low Back Pain':ab,ti OR 'Low Back Pain, Mechanical':ab,ti OR 'Mechanical Low Back Pain':ab,ti: 6,785 results

#6 Search 'low back pain'/exp OR 'Back Pain, Low':ab,ti OR 'Back Pains, Low':ab,ti OR 'Low Back Pains':ab,ti OR 'Pain, Low Back':ab,ti OR 'Pains, Low Back':ab,ti OR 'Lumbago':ab,ti OR 'Lower Back Pain':ab,ti OR 'Back Pain, Lower':ab,ti OR 'Back Pains, Lower':ab,ti OR 'Lower Back Pains':ab,ti OR 'Pain, Lower Back':ab,ti OR 'Pains, Lower Back':ab,ti OR 'Low Back Ache':ab,ti OR 'Ache, Low Back':ab,ti OR 'Back Ache, Low':ab,ti OR 'Aches, Low Back':ab,ti OR 'Back Aches, Low':ab,ti OR 'Low Back Aches':ab,ti OR 'Low Backache':ab,ti OR 'Backache, Low':ab,ti OR 'Backaches, Low':ab,ti OR 'Low Backaches':ab,ti OR 'Low Back Pain, Postural':ab,ti OR 'Postural Low Back Pain':ab,ti OR 'Low Back Pain, PosteriOR Compartment':ab,ti OR 'Low Back Pain, Recurrent':ab,ti OR 'Recurrent Low Back Pain':ab,ti OR 'Low Back Pain, Mechanical':ab,ti OR 'Mechanical Low Back Pain':ab,ti: 57,019 results

#7 Search 'randomized controlled trial':ab,ti or 'randomized':ab,ti or 'placebo':ab,ti: 859366 results

#8 Search #6 AND #7 AND #8 AND ([controlled clinical trial]/lim OR [randomized controlled trial]/lim): 13 results


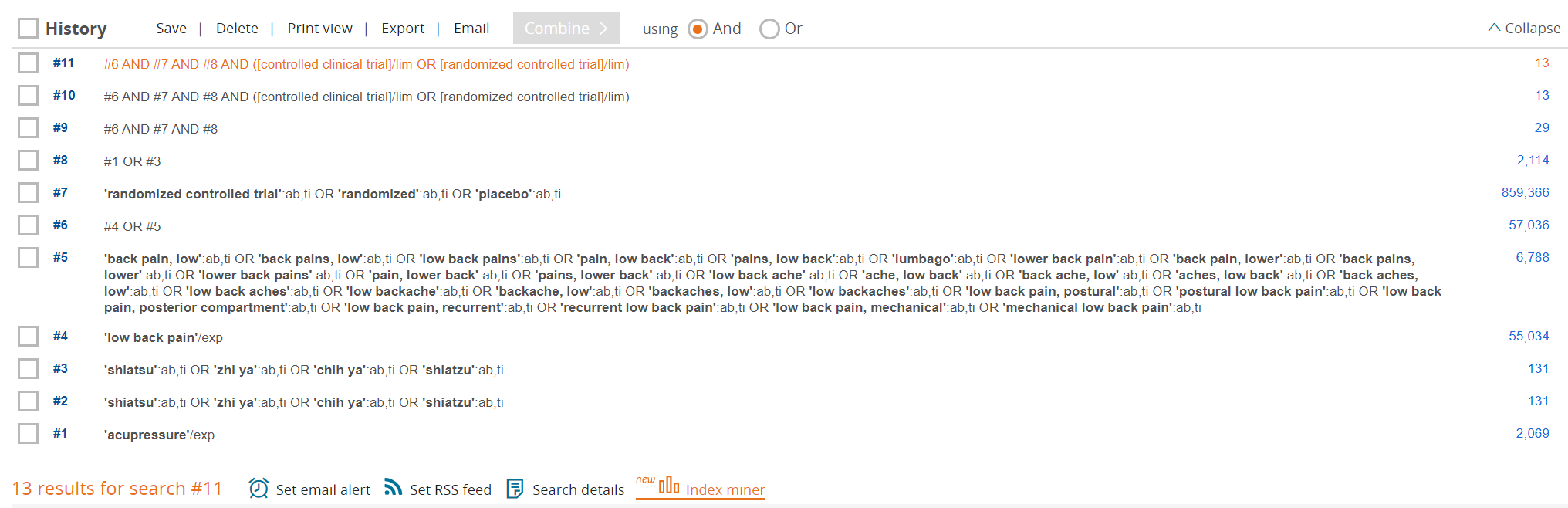


# 3. Searching strategy in Cochrane Library

# 3.1 Steps and details of literature search in Cochrane Library

# #1: MeSH: acupressure: 319 results

# #2: MeSH: (Shiatsu): ti,ab,kw OR (Zhi Ya):ti,ab,kw OR (Chih Ya):ti,ab,kw OR (Shiatzu):ti,ab,kw: 24 results

# #3: MeSH: (Acupressure):ti,ab,kw OR (Shiatsu):ti,ab,kw OR (Zhi Ya):ti,ab,kw OR (Chih Ya):ti,ab,kw OR (Shiatzu):ti,ab,kw: 1229 results

# #4: MeSH: (Low back pain):ti,ab,kw OR (Back Pain, Low):ti,ab,kw OR (Back Pain, Low):ti,ab,kw OR (Back Pain, Low):ti,ab,kw OR (Back Pain, Low):ti,ab,kw: 10116 results

# #5: MeSH: (randomized controlled trial):ti,ab,kw OR (randomized):ti,ab,kw OR (placebo):ti,ab,kw: 905493 results

# #6: #3 AND #4 AND #5: 42 results


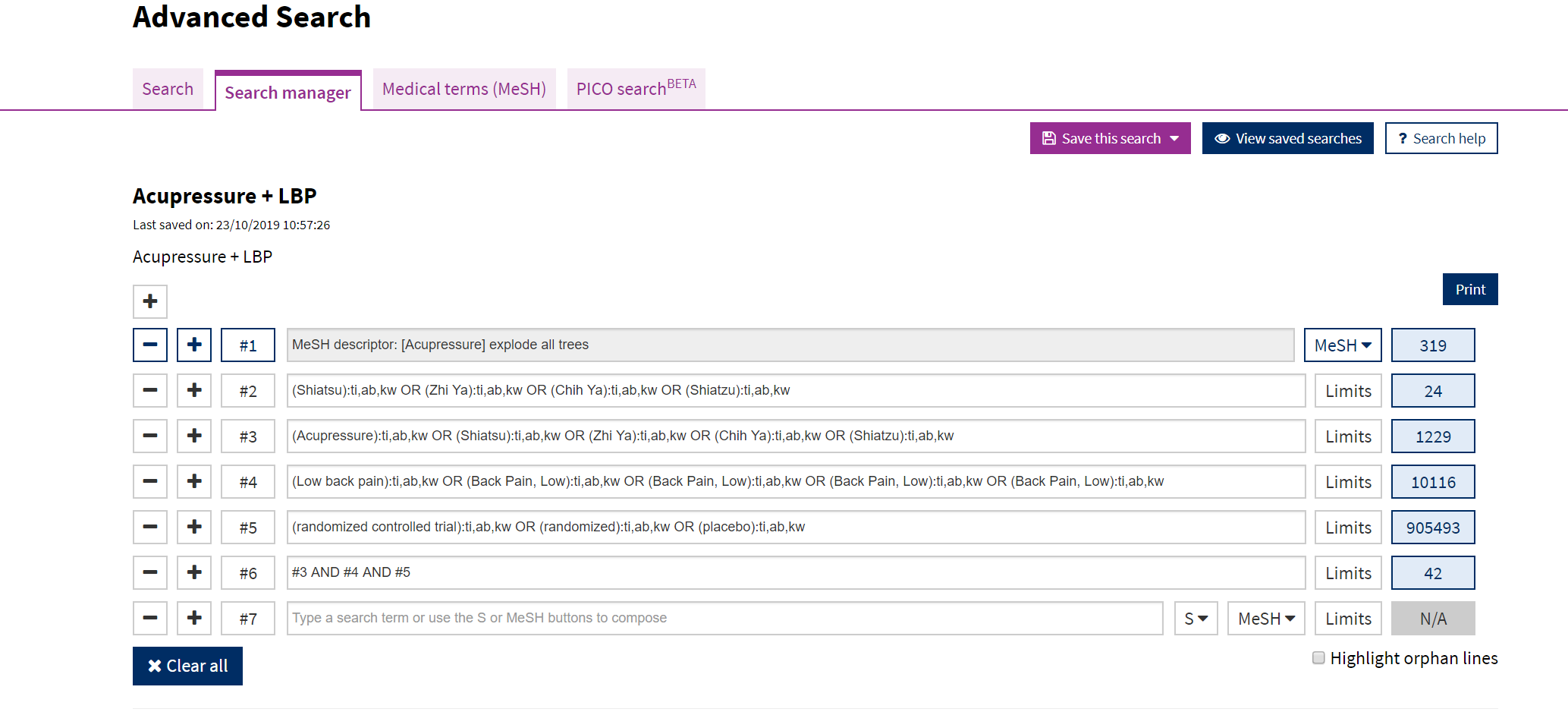


# 4. Searching strategy in Clinical Registry.gov

#1 Condition or disease: low back pain

# #2 Intervention/treatment: Acupressure or Shiatsu or Zhi Ya or Chih Ya or Shiatzu

#3 Study type: Intervention Studies (Clinical Trials)

#4 Study Results: All Studies

Results: 8


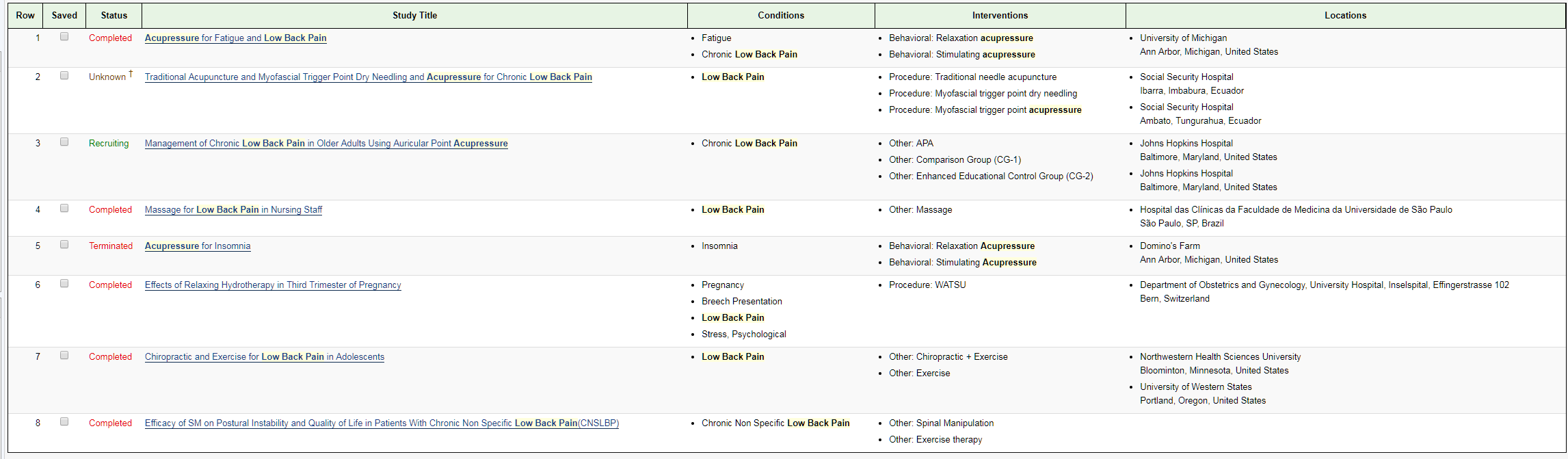


# For the searching strategy in Chinese electronic database. We used the Chinese translation of MeSH. The Chinese translation of MeSH was shown as follows: "lumbar disc herniation: *yao zhui jian pan tu chu*", "lumbar stenosis: *yao zhui guan xia zhai*", "lumbar spondylolisthesis: *yao zhui hua tuo*", "the third transverse process syndrome: *di san heng tu zong he zheng*", " lumbar muscle strain: *yao ji lao sun*", "low back pain: *xia yao tong*", “sciatica: *yao tui tong*”, “low back pain: *xia yao tong*”, “lumbago: *yao tong*”, “lumbago: *xia yao tong*”, “acupressure: *dian xue*”, “acupressure/shiatsu: *zhi ya*”, “acupoint or acupuncture point: *xue wei an ya*”, “acupressure: *dian an*”, “acupressure/finger needle: *zhi zhen*”, “randomized control*sui ji dui zhao*”, “randomization: *sui ji*”, “control: *dui zhao*”, “randomized trials: *sui ji shi yan*”.

# 5. Searching strategy in CNKI

#1 Disease (title)：*yao bei teng tong*, *yao zhui jian pan tu chu*, *yao zhui hua tuo*,, *di san heng tu zong he zheng*, *yao ji lao sun*, *yao zhui guan xia zhai*, *yao tui tong, xia yao tong*, *yao tong*, *yao bei tong*

Results: 98,718 results

#2 Intervention (title, search in previous results)：*dian xue*, *zhi ya*, *xue wei an ya*, *dian an*.

Results: 267 results

#3 Design (abstract，search in previous results)：*sui ji dui zhao*, *sui ji, dui zhao*, *RCT*.

Results: 64 results

6. Searching strategy in VIP

#1 Disease (title)：*yao bei teng tong + yao zhui jian pan tu chu + yao zhui hua tuo + + di san heng tu zong he zheng + yao ji lao sun + yao zhui guan xia zhai + yao tui tong+ xia yao tong + yao tong + yao bei tong*

Results: 51,786 results

#2 Intervention (title, search in previous results)：*dian xue* + *zhi ya* + *xue wei an ya* + *dian an*

Results: 112 results

#3 Design (abstract, search in previous results)：*sui ji dui zhao + sui ji + dui zhao + RCT*

Results: 37 results

7. Searching strategy in Wanfang Database

#1 Disease (title)：*yao bei teng tong + yao zhui jian pan tu chu + yao zhui hua tuo + + di san heng tu zong he zheng + yao ji lao sun + yao zhui guan xia zhai + yao tui tong+ xia yao tong + yao tong + yao bei tong*

Results: 49203 results

#2 Intervention (title, search in previous results)：*dian xue + zhi ya+xue wei an ya + dian an*

Results: 107 results

#3 Design (abstract, search in previous results)：*sui ji dui zhao + sui ji + dui zhao + RCT*

Results: 43 results

8. Searching strategy in CBM

#1 Disease (title)："*yao zhui jian pan tu chu*" [Commonly used fields: intelligence] OR "*yao bei tong*" [Commonly used fields: intelligence] "*xia yao tong*" [Commonly used fields: intelligence] OR "*xia yao tong*" [Commonly used fields: intelligence] OR "*yao zhui hua tuo*" [Commonly used fields: intelligence] OR "*di san heng tu zong he* *zheng*" [Commonly used fields: intelligence] OR "*yao tui tong*" [Commonly used fields: intelligence] OR "*yao ji lao sun*" [Commonly used fields: intelligence] OR "*yao zhui jian pan tu chu*" [Commonly used fields: intelligence] OR "*yao tong*"[Unweighted: extended]

Results: 58403 results

#2 Intervention (title, search in previous results)： ["*dian an*" [Commonly used fields: intelligence] OR "*xue wei an ya*" [Commonly used fields: intelligence] OR "*zhi ya*" [Commonly used fields: intelligence] OR "*dian xue*" [Unweighted: extended]](javascript:toDoRelimitSearch();)

Results: 10413 results

#3 Research（Abstract）： "*sui ji dui zhao*" [Abstract: intelligence] OR "*sui ji*" [Abstract: intelligence] OR "*dui zhao*"[Abstract: intelligence] OR "*RCT*" [Abstract: intelligence]

Results: [2293356](javascript:void(0);) results

#4: (#1) AND (#2) AND (#3)

Results: 122 results

# 9. Searching strategy in Chinese Clinical Trial Registry

#1 Condition or disease: *low back pain* + *yao bei teng tong + yao zhui jian pan tu chu + yao zhui hua tuo + di san heng tu zong he zheng + yao ji lao sun + yao zhui guan xia zhai + yao tui tong+ xia yao tong + yao tong + yao bei tong*

# #2 Intervention/treatment: *dian xue + zhi ya + xue wei an ya + dian an*

Study type: no limit;

Study Results: no limit;

Results: 0


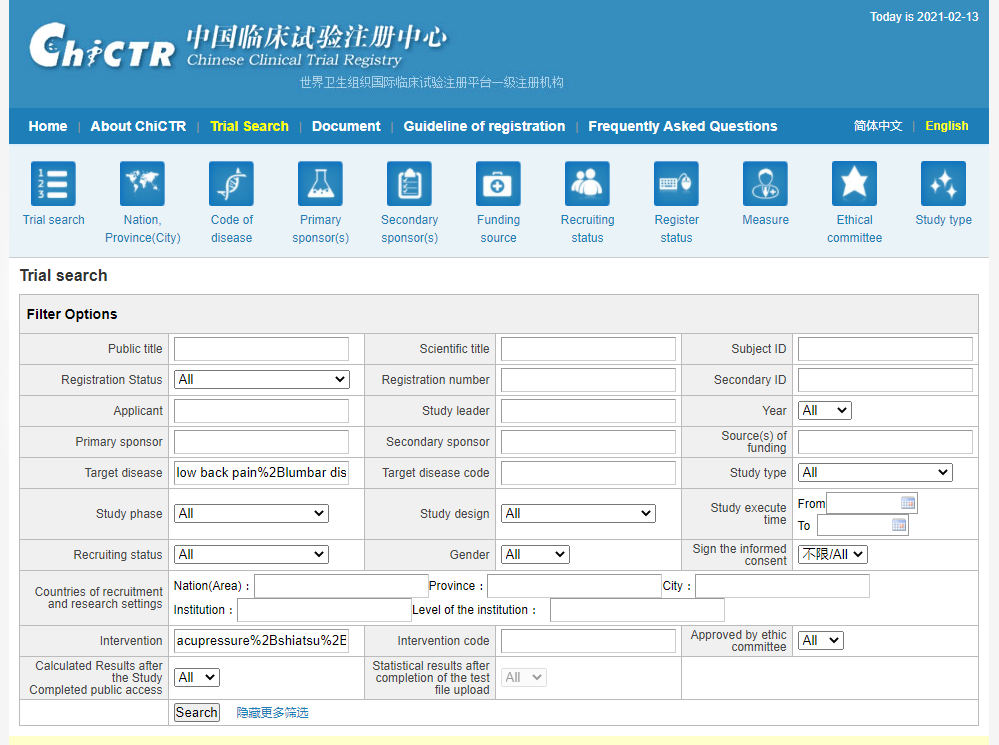


10. Grey Literature Searching

Web site: <http://opengrey.eu/>

MeSH: acupressure LBP OR acupressure low back pain

Result：0
